# Lipofundin-Induced Hyperlipidemia Promotes Oxidative Stress and Atherosclerotic Lesions in New Zealand White Rabbits

**DOI:** 10.1155/2012/898769

**Published:** 2011-09-29

**Authors:** Livan Delgado Roche, Emilio Acosta Medina, Ángela Fraga Pérez, María A. Bécquer Viart, Yosdel Soto López, Viviana Falcón Cama, Ana M. Vázquez López, Gregorio Martínez-Sánchez, Eduardo Fernández-Sánchez

**Affiliations:** ^1^Center of Studies for Research and Biological Evaluations, Pharmacy and Food Science College, University of Havana, PO. Box 13 600, La Coronela, La Lisa, Havana 13600, Cuba; ^2^Department of Antibody Engineering, Center of Molecular Immunology, Havana 11600, Cuba; ^3^Department of Electron Microscopy, Center for Genetic Engineering and Biotechnology, Havana 10600, Cuba; ^4^MediNat, 60021 Ancona, Italy

## Abstract

Atherosclerosis represents a major cause of death in the world. It is known that Lipofundin 20% induces atherosclerotic lesions in rabbits, but its effects on serum lipids behaviour and redox environment have not been addressed. In this study, New Zealand rabbits were treated with 2 mL/kg of Lipofundin for 8 days. Then, redox biomarkers and serum lipids were determined spectrophotometrically. On the other hand, the development of atherosclerotic lesions was confirmed by eosin/hematoxylin staining and electron microscopy. At the end of the experiment, total cholesterol, triglycerides, cholesterol-LDL, and cholesterol-HDL levels were significantly increased. Also, a high index of biomolecules damage, a disruption of both enzymatic and nonenzymatic defenses, and a reduction of nitric oxide were observed. Our data demonstrated that Lipofundin 20% induces hyperlipidemia, which promotes an oxidative stress state. Due to the importance of these phenomena as risk factors for atherogenesis, we suggest that Lipofundin induces atherosclerosis mainly through these mechanisms.

## 1. Introduction

Atherosclerosis is a chronic vascular disease and a leading cause of death in the western world. It is well established that hyperlipidemia and oxidative stress (OS) are major contributors to atherogenic development [1]. The retention of low-density lipoproteins (LDL) in the arterial wall [2] and their oxidation by reactive oxygen species (ROS) initiates a complex series of biochemical and inflammatory reactions [3, 4]. Oxidized LDL (ox-LDL) are internalized by macrophages through the scavenger receptors, leading to foam cell formation [5]. Furthermore, oxidized cholesterol products present in blood and in arterial plaques increase cholesterol biosynthesis, affect plasma membrane structure, cell proliferation, and cell death, and promotes atherosclerosis development [6].

The rabbit is one of the most widely used animal models in atherosclerosis research. One strategy to induce atherosclerotic lesions in these animals is through an intravenous administration of Lipofundin 20%, a lipid-rich emulsion used in parenteral nutrition, which produces aortic lesions, characterized by subendothelial lipid accumulation, intimal thickening, and a distortion of vascular tissue architecture [7, 8]. The impact of Lipofundin 20% administration on lipid levels and redox environment in New Zealand white (NZW) rabbits had not been studied. In the present work, we demonstrated that Lipofundin 20% induces a hyperlipemic state and a systemic/aortic oxidative stress, which can lead to atherosclerotic lesions development. 

## 2. Materials and Methods

### 2.1. Animals

Standard NZW male rabbits, weighing 2.0–2.5 kg and 12 weeks old, were obtained from CENPALAB (Bejucal, Havana, Cuba). Rabbits were housed under conventional conditions exposed to light-dark cycle of 12 h with free access to water and food. Animal studies were performed with the approval of Pharmacy and Food Sciences College Institutional Animal Ethical Committee. All procedures were performed in accordance with the guidelines stipulated by the Institutional Animal Care Committee and the European Union Guidelines for animal experimentation.

### 2.2. Lipofundin Composition

Lipofundin MCT/LCT 20% (Braun Melsungen AG, Melsungen, Germany) is a lipid emulsion containing soya oil 100 g, medium-chain triglycerides 100 g, glycerol 25 g, egg lecithin 12 g, *α*-tocopherol 170 ± 40 mg, and sodium oleate/water for injection in sufficient quantity to 1000 mL.

### 2.3. Experimental Design

Two groups of 10 rabbits were used in the study. The first group received an intravenous injection of phosphate-buffered saline (PBS), pH 7,4 (control group), and the second one received a slow intravenous injection of 2 mL/kg of Lipofundin MCT/LCT 20%, as an infusion during 1-2 min [7, 8]. This procedure was repeated daily during a period of 8 days. On day 9, the animals were anesthetized with ketamine hydrochloride (5 mg/kg i.m.) and euthanized with an overdose of sodium pentobarbital (90 mg/kg, i.v.). (Abbott Laboratories, Mexico SA de CV, Mexico), and the vascular system was perfused with NaCl 0.9% solution at 4°C. Then, aortas were excised from the aortic arch to abdominal segment, and adventitial fat was removed. Aortic arches were used for histopathology and redox evaluations due to the preferential development of Lipofundin 20%-induced atherosclerotic lesions in this segment [8]. For each evaluation, the samples of five animals per group were used. 

### 2.4. Serum Sample Collection

Blood samples (3 mL) were obtained on day 0 (before Lipofundin administration) and on day 9 (at the end of the study), for biochemical analyses. Blood was withdrawn from the rabbit's marginal ear vein. These samples were immediately centrifuged at 2500 g, at 4°C for 10 min. The serum was collected and aliquots were stored at −80°C until analysis.

### 2.5. Aortic Homogenate Preparation

Aortic arches were placed in ice-cold 0.1 mol/L Tris-HCl buffer, pH 7,6 containing 1.0 mmol/L EDTA and 0.2 mmol/L butylated hydroxytoluene (buffer A) and macerated before homogenization in a tissue homogenizer (Edmund Bühler LBMA, Germany). Homogenized tissue was then centrifuged at 4500 g for 20 min at 4°C, and the supernatants were collected and stored at −80°C until redox biomarkers determinations.

### 2.6. Histopathology

#### 2.6.1. Eosin-Hematoxylin Staining

Aortic arches were rinsed in PBS, pH 7,4, transversally cut, and fixed in 10% formaldehyde solution. Samples were then embedded in paraffin. Five-micrometer tissue sections were cut, air-dried on glass slides, deparaffinized, and rehydrated. Finally, tissue sections were stained with eosin and hematoxylin (HE) under standard procedures. The sections were analyzed in an optic microscope Olympus BX51.

### 2.7. Ultrastructural Analysis

#### 2.7.1. Electron Transmission Microscopy

For transmission electron microscopy (TEM), samples from rabbit aortic arch were fixed for 1 h at 4°C in 3.2% glutaraldehyde (Agar Scientific, UK), 0.1 M phosphate buffer (pH 7,4) and postfixed in 1% OsO_4_ for 1 h. After graded ethanol dehydration, samples were embedded in Spurr low-viscosity epoxy resin for 24 h at 37°C. Ultrathin sections were cut into 400–500 Å thick slice with an ultramicrotome (NOVA, LKB), counterstained with uranyl acetate and lead citrate, and analyzed in a TEM (JEOL JEEM-2000EX, JEOL, Japan).

#### 2.7.2. Serum Lipid Assay

Serum total cholesterol, triglycerides, LDLc, and HDLc were determined using commercial enzymatic kits (Randox, Crumlin, UK).

#### 2.7.3. Redox Biomarkers Determinations

All biochemical parameters were determined by spectrophotometric methods using a Pharmacia 1000 Spectrophotometer (Pharmacia LKB, Uppsala, Sweden). Total proteins levels were determined using the method described by Bradford [9] with bovine serum albumin as standard. SOD activity was determined by using RANSOD kit (catalogue no. SD 125, Randox Labs, Crumlin, UK), where xanthine and xanthine oxidase were used to generate superoxide anion radicals (O_2_^•−^), which react with 2-(4-iodophenyl)-3-(4-nitrophenol)-5-phenyltetrazolium chloride (INT) to form a red formazan dye. SOD activity was measured by the inhibition degree of this reaction. Catalase (CAT) activity was determined by following the decomposition of hydrogen peroxide (H_2_O_2_) at 240 nm at 10 s intervals during 1 min [10]. 

After precipitation of thiol proteins, the reduced glutathione (GSH) levels were measured according to the method of Sedlak and Lindsay [11] with Ellman's reagent (5,5′dithiobis-2-nitrobenzoic acid) (Sigma St. Louis, MO, USA), and the absorbance was measured at 412 nm. Purified GSH (Sigma St. Louis, MO, USA) was used to generate standard curves. 

The advanced oxidation protein products (AOPPs) were measured as described previously [12]. Briefly, samples in PBS (1 mL) were treated with 50 *μ*L of potassium iodide 1.16 M followed by the addition of 100 *μ*L of acetic acid. The absorbance was immediately read at 340 nm. AOPP concentration was expressed as *μ*M of chloramines-T. 

Concentration of malondialdehyde (MDA) was determined using the LPO-586 kit obtained from Calbiochem (La Jolla, CA, USA). In the assay, the production of a stable chromophore after 40 min of incubation at 45°C was measured at 586 nm. For standards, freshly prepared solutions of malondialdehyde bis (dimethyl acetal) (Sigma St. Louis, MO, USA) were employed and assayed under identical conditions [13, 14]. 

In order to determine susceptibility to lipid peroxidation and total reactive antioxidant power (TRAP), the samples were incubated with a solution of copper sulphate (final concentration 2 mM) at 37°C for 24 h. The peroxidation potential (PP) was calculated by subtracting the MDA levels before the induction of lipid peroxidation from the one obtained at 24 h [15]. 

Nitrites (NO_2_^−^) level, as a surrogate marker of nitric oxide (NO^•^), were determined converting nitrates to nitrites using nitrate reductase (Boehringer Mannheim Italy SpA, Milan, Italy). Then, Griess reagent (1% sulphanilamide, 0.1% N-(1-Naphthyl)-ethylenediamine dihydrochloride in 0.25% phosphoric acid) was added [16]. Samples were incubated at room temperature for 10 min, and absorbance was measured at 540 nm.

### 2.8. Statistical Analysis

Statistical analysis was performed using the SPSS program for Windows (version 11.5, SPSS Inc). Bartlett's Box-test was used to test the homogeneity of variance. Differences between groups were determined by student's *t*-test (two-tailed). Data were expressed as the mean ± standard deviation (SD). A *P* value of < 0.05 was considered statistically significant. 

## 3. Results

### 3.1. Histopathology

**Figure 1:**
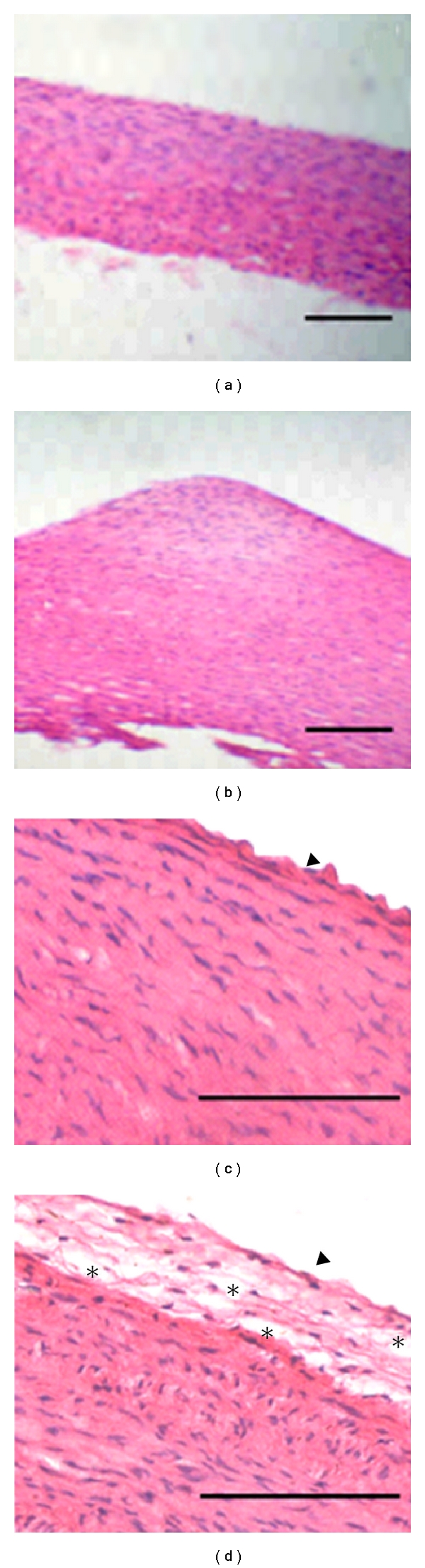
Histopathological analysis of rabbit's aortic tree. Eosin/hematoxylin staining reveals a normal morphology of aortas in control animals (a) and (c), while aortas of Lipofundin group show an intimal thickening, characterized by a vascular tissue architecture distortion and large extracellular spaces, probably filled with lipids (asterisks) (b) and (d). Arrow head: endothelial cells. Magnification 10× (a) and (b) and 40× (c) and (d). Scale bar, 20 *μ*m.

The HE staining of aortic arch sections from control rabbits showed neither intimal thickening nor distortion in the vascular tissue architecture (Figures 1(a) and 1(c)). In contrast, aortic sections from those animals who received intravenously 2 mL/kg of Lipofundin 20% during 8 days showed a thickening of the intima with apparent lipid accumulation and distortion of tissue architecture (Figures 1(b) and 1(d)). 

Nonrelevant disease or abnormalities in other organs were detected by macroscopic and microscopic examination.

### 3.2. Ultrastructural Analysis

**Figure 2:**
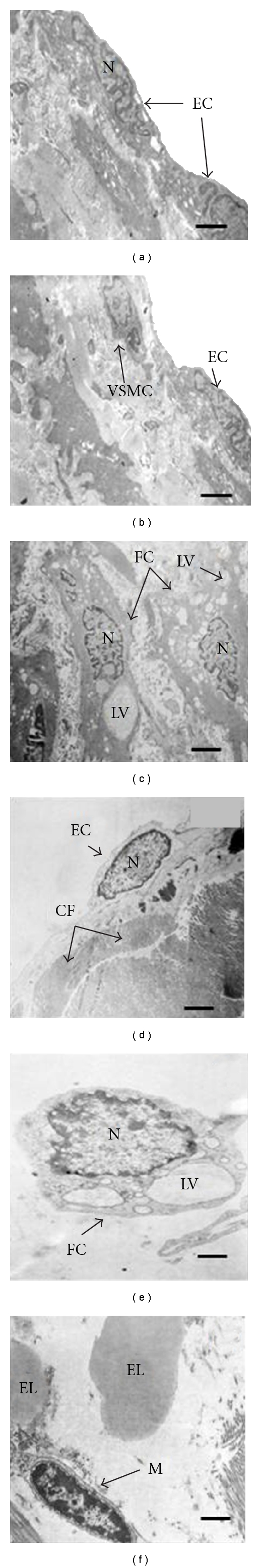
Ultrastructural analysis. Panels (a) and (b) correspond to animals of control group, while panels (b), (c), (d), and (e) show the effects of Lipofundin administration on atherosclerotic lesion formation. EC: endothelial cells, EL: extracellular lipids, VSMC: vascular smooth muscle cells, N: nucleus, FC: foam cells, LV: lipid vacuolization, CF: collagen fibers, M: myofibroblasts. Scale bar 1 *μ*m (a, b, c, d), 500 nm (e, f).

On the other hand, the ultrastructural analysis confirmed the results observed by light microscopy. In the animals treated with Lipofundin was observed an endothelial damage characterized by a loss of endothelium integrity and the presence of abundant foam cells and myofibroblasts in the intima and media layers. Also, we observed a high extracellular lipid accumulation and collagen fibers deposition (Figures 2(c), 2(d), 2(e), 2(f)). No alterations in the aortic artery wall of control rabbits were observed (Figures 2(a) and 2(b)).

### 3.3. Serum Lipids

Serum total cholesterol, triglycerides, LDLc, and HDLc levels showed a significant increase (*P* < 0.05) in those animals who were treated during 8 days with the lipid-rich emulsion Lipofundin, while no significant changes in serum lipids were observed in the control rabbits throughout the study (Table 1).

### 3.4. Redox Biomarkers

Table 2 shows the behavior of serum and aortic redox parameters in both groups. The biomolecules damages markers were significantly (*P* < 0.05) modified after 8 days of Lipofundin administration compared to nontreated group. At the end of the experimental period, the MDA levels, one of the end-products of lipid peroxidation, were higher in Lipofundin-treated animals compared with controls. Besides, Lipofundin treatment also caused a rise of AOPP levels in comparison with control group. The activity of both antioxidant enzymes SOD and CAT were significantly higher (*P* < 0.05) in Lipofundin group at the end of the experiment compared to control rabbits. The NO_2_^−^ levels and GSH concentration decreased significantly after 8 days of Lipofundin treatment in comparison to those of untreated animals (*P* < 0.05). Finally, the susceptibility to lipid peroxidation was higher in those animals who received Lipofundin. After 8 days, in these animals was observed a significant increase of PP (*P* < 0.05), compared to the one calculated in controls.

## 4. Discussion

The histopathological analyses of the aortic sections from rabbits treated with Lipofundin 20% demonstrated the capacity of Lipofundin to induce atherosclerotic lesions. As described above, an intimal thickening and a distortion of tissue architecture was observed by EH staining. Electron microscopy confirmed the presence of foam cells, extracellular lipid accumulation, collagen fibers deposition, vascular smooth muscle cells (VSMC) migration, the presence of myofibroblasts, and also the loss of endothelium integrity. These events, induced by Lipofundin 20%, contribute with the development and progression of atherosclerosis.

At the end of the experiment, we observed high serum levels of triglycerides, total cholesterol, LDLc, and HDLc in the animals treated with Lipofundin 20% in comparison to control rabbits. Indeed, there is a causal relationship between the elevated plasma lipids and the development of atherosclerotic lesions [17–19]. 

Lipofundin 20%-induced hyperlipidemia could be associated with the high content of triglycerides in this emulsion. High levels of exogenous triglycerides promote ApoB100 and cholesterol synthesis and eventually the assembly of very low-density lipoproteins (VLDL) [20]. In fact, Lipofundin 10% caused a 60% increase in total serum cholesterol after parenteral administration in a human study [21].

In addition, there is a mutual exchange of lipids and apolipoproteins between serum lipoproteins and the infused triglyceride/phospholipid particles [22]. The increase of HDLc may be determined by a physiological response against the elevated LDLc levels. It is known that HDL protect from atherosclerotic development. However, based on recent animal and epidemiological studies, it appears that in addition to quantity [23] other properties of HDL, such as antioxidant and anti-inflammatory power, are necessary for atheroprotection [24, 25].

In this study, we demonstrated that Lipofundin-induced hyperlipidemia was associated with a systemic and aortic OS. Strong evidences for the involvement of free radicals production in the onset of hyperlipidemia have been reported previously [26]. Chronic generation and sustained high toxic levels of ROS are associated with several pathological conditions including cardiovascular diseases such as atherosclerosis [27]. During atherosclerotic lesions development, cellular damages take place through mechanisms involving lipid peroxidation and oxidative modifications of proteins [28]. On the other hand, a disruption of antioxidant enzymes activity and a drastic reduction of nonenzymatic defenses are also observed during atherogenesis [29]. High levels of MDA in the sera and aortic tissue from rabbits bearing atherosclerotic lesions, compared with those from control group, suggest the role of LPO in the loss of redox cellular status in the former animals which were under atherogenic stimuli caused by Lipofundin treatment. MDA levels have been considered not only an indicator of OS, but also as a biochemical marker of atherogenesis [30, 31].

Oxidative modifications of proteins have been also implicated in atherosclerosis [32]. Through AOPP determination, we measured the chlorinated proteins levels, caused by myeloperoxidase-derived hypochlorous acid (HOCl). It has been shown that HOCl-modified proteins are present in atherosclerotic lesions and predict the progression of the disease [5]. The high levels of AOPP in those animals that received the lipid emulsion suggest an active role of macrophages infiltration and inflammatory process in the development of atherosclerotic lesions in the present animal model.

Antioxidant defenses, as expression of the balance between generation and inactivation of oxidized metabolites, represent a useful tool to examine the redox status [33, 34]. In our study, the higher activity of extracellular SOD, detected in the animals treated with Lipofundin, could be associated with an increase in O_2_^•−^ generation, typically produced by foam cells and macrophages at atherosclerotic lesion sites [35]. Also, in atherogenic process, there is an increase in vascular NADPH oxidase activity, the main source of O_2_^•−^ in the vasculature [36].

CAT is another antioxidant enzyme present in the vasculature, which plays an important role on redox environment maintenance [37]. In our study, we found a high activity of the enzyme in animals treated with Lipofundin. During the beginning and development of atherogenic lesions, the enzyme gene expression increases and in this way contributes to retard the disease progression [38, 39]. Also, it has been shown that in early steps of atherogenesis CAT activity is incremented in response to oxidant stimulus mediated by ox-LDL and ROS such as H_2_O_2_ and lipoperoxides [40].

During atherogenesis, the reactive molecules that are produced have the potential to deplete the surrounding cells of their GSH levels, affecting their antioxidant defenses and detoxification pathways [41]. Our results showed a significant depletion of serum and aortic GSH levels in the animals treated with the lipid emulsion compared to the control rabbits. This fact could be associated with the Lipofundin-mediated ROS generation and with the high concentration of biomolecules damages detected in Lipofundin-treated animals.

Finally, we evaluated the behaviour of NO_2_^−^ levels, as a marker of NO^•^ bioavailability. NO^•^ is a vasoactive molecule which has an important role in vascular homeostasis maintenance [42]. The decrease of NO^•^ bioavailability is considered an important indicator of vascular endothelial dysfunction contributing to atherosclerosis development [43]. Our experimental results showed a reduced bioavailability of NO^•^ in Lipofundin-treated rabbits compared with controls. This deleterious effect for vascular function may contribute with the Lipofundin-induced atherogenic development.

## 5. Conclusions

In summary, the present study demonstrated that Lipofundin 20% induces hyperlipidemia, thereby promoting a systemic and aortic OS and also contributing with atherosclerotic lesions formation in NZW rabbits. This work shows novel evidences of Lipofundin-induced oxidative damages on lipids and proteins, the impairment of antioxidant status, and the reduction of nitric oxide levels. These results reinforce the attractive characteristics of Lipofundin to be used as an inductor of experimental atherosclerosis in rabbits. The reduction of experimental time and the associated costs, compared with other established models, is in our opinion the main advantage of this animal model of atherosclerosis.

##  Conflict of Interests

There are not conflict of interests.

## Figures and Tables

**Table 1:** Effects of Lipofundin on serum lipid profile. Values represent the mean ± standard deviation. Asterisks represent statistical differences (*P* < 0.05).

	Control	Lipofundin
TC, mmol/L	1.78 ± 0.06	3.10 ± 0.13*
TG, mmol/L	1.51 ± 0.03	2.73 ± 0.07*
HDLc, mmol/L	0.76 ± 0.04	1.20 ± 0.04*
LDLc, mmol/L	0.18 ± 0.01	0.83 ± 0.03*

**Table 2:** Effects of Lipofundin on redox biomarkers. Values represent the mean ± standard deviation. Asterisks represent statistical differences (*P* < 0.05). The concentration of aortic parameters is expressed per milligrams of total proteins (Pr).

	Control	Lipofundin
Systemic redox biomarkers		

MDA, *μ*M	2.69 ± 0.07	6.24 ± 0.28*
AOPP, *μ*M of chloramines	11.50 ± 0.73	16.22 ± 0.47*
PP, *μ*M of MDA	4.63 ± 0.18	9.13 ± 0.34*
CAT, U/L/min	351.13 ± 19.03	477.50 ± 30.46*
SOD, U/mL/min	22.03 ± 26.44	32.00 ± 1.60*
NO_2_, *μ*M	179.18 ± 11.44	134.33 ± 5.09*
GSH, *μ*M	309.03 ± 26.44	191.21 ± 8.26*

Aortic redox biomarkers		

MDA, *μ*M/mgPr	18.49 ± 2.04	27.42 ± 2.55*
AOPP, *μ*M of chloramines/mgPr	12.45 ± 1.21	24.25 ± 1.86*
PP, *μ*M of MDA/mgPr	13.81 ± 1.83	25.26 ± 2.29*
CAT, U/L/min/mgPr	1023.60 ± 26.89	1609.68 ± 84.37*
SOD, U/mL/min/mgPr	62.37 ± 3.93	105.39 ± 9.82*
NO_2_, *μ*M/mgPr	95.29 ± 2.54	43.96 ± 6.03*
GSH, *μ*M/mgPr	166.70 ± 12.82	71.59 ± 10.89*
